# Assessment of the yellow fever outbreak in Angola from December 2015 through December 2016: A retrospective study

**DOI:** 10.1002/hsr2.1924

**Published:** 2024-02-15

**Authors:** Eusebio Manuel, António Armando, Moisés Francisco, Joana Paixão, Javier Aramburu, Miguel dos Santos de Oliveira, Helga Freitas, Alda Morais Pedro, Domingos Jandondo, Pablo Babrero Carderon, Sandra Lopez Lamezon, Filomeno Fortes, Jorge Mariscal, Yolanda Cardoso, Rosa Moreira, Joana Morais, Ngiambudulu M. Francisco

**Affiliations:** ^1^ Faculdade de Medicina Universidade Agostinho Neto Luanda Angola; ^2^ Direcção Nacional de Saúde Pública Ministério da Saúde Luanda Angola; ^3^ Grupo de Investigação Microbiana e Imunológica Instituto Nacional de Investigação em Saúde (National Institute for Health Research) Luanda Angola; ^4^ World Health Organization (OMS, Angola) Luanda Angola; ^5^ Clínica Girassol Luanda Angola; ^6^ Instituto de Higiene e Medicina Tropical Universidade Nova de Lisboa Lisboa Portugal

**Keywords:** *Aedes aegypti*, Angola, flavivirus, infectionyellow fever

## Abstract

**Background and Aims:**

The acute tropical infectious disease known as yellow fever (YF) is caused by an arbovirus and is characterized by fever, jaundice, hemorrhage, headache, muscle pain, nausea, vomiting, and fatigue. Angola experienced a yellow fever virus (YFV) outbreak that was documented in December 2015. However, little is known about the outcome of this outbreak. We aimed to demonstrate epidemic features and lessons learned during the YF epidemic in Angola.

**Methods:**

A total of 4618 blood samples from suspected YF cases were sent to the Instituto Nacional de Investigação em Saúde (INIS), a national referral and public health laboratory, between December 5, 2015, and December 23, 2016. Sample analyses were conducted using enzyme‐linked immunosorbent assay (ELISA) and reverse transcription polymerase chain reaction (RT‐PCR) assays. Blood samples were sent from 16 out of the 18 provinces of Angola.

**Results:**

We detected 884 (19.1%) cases that were positive for ELISA, which were confirmed by RT‐PCR assay. Considering the positive cases, the incidence among male patients was around three times higher (*n* = 223; 10.9%) than in female patients (*n* = 59; 2.6%) in the 20–29 age group, followed by the age group 10–19 with *n* = 211 (6.8%) in males versus *n* = 108 (3.3%) in females; and the age group 30–39 had *n* = 68 (4.8%) in males versus *n* = 28 (1.8%) in females. The other groups had an incidence below 3.0%. The case fatality ratio for YF was in young adults in the age group 20–29 with *n* = 39 cases, followed by the age group 10–19 with *n* = 16 cases, and finally the age group 0–9 with *n* = 13 cases. The other age groups had several deaths by YF below 10 cases.

**Conclusions:**

This study demonstrates features of the YF epidemic that occurred in Angola. Also, it demonstrates that YF causes deaths in young people but is preventable by high vaccine coverage. Thus, public health laboratory surveillance must be strengthened to reduce the possibility of emerging and re‐emerging human infections.

## 1 INTRODUCTION

Yellow fever (YF) is a viral infection caused by the yellow fever virus (YFV) of the flavivirus genus, this genus includes Japanese encephalitis, West Nile, Dengue, tick‐borne encephalitis viruses, and are all transmitted primarily by the bite of an infected *Aedes aegypti* mosquito.^1^ The *A. aegypti* mosquito is a species that is commonly found in tropical and subtropical around the world. Infection with YFV can result in subclinical to severe illness, characterized by jaundice, fever, headache, hemorrhage, muscle pain, nausea, vomiting, and fatigue.^2^ Scientific evidence has shown that the clinical course of this disease is traditionally divided into three stages: (i) infection, (ii) remission, and (iii) intoxication.^3^, ^4^, ^5^ Intoxication happens when symptoms reappear and has been considered the severe form of YF.^3^ The YFV is endemic in the following tropical regions: (a) Central and South America, and (b) Africa, where 46 countries and almost 1 billion people are at risk. YF was initially identified in Angola in the 1930s, but no significant outbreaks were reported until 1971 (65 cases) and 1988 (37 cases).^6^, ^7^, ^8^ The outbreak of YF in Angola occurred in December 2015 and continued until December 2016, despite the nation having distributed close to 12 million vaccination doses. Alarmingly, in the year 2016, the Angolan YF outbreak spread to Kinshasa, the Democratic Republic of the Congo, with greater international spread beyond Angola to other nations, including Kenya and China, highlighting the difficulty of managing infectious diseases in this historical era of extraordinary mobility.^2^, ^9^

YFV has three transmission cycles: jungle, intermediate, and urban. These cycles occur as follows: (i) The jungle transmission cycle, also known as the sylvatic cycle, is characterized by monkeys (nonhuman primates' species) and mosquitoes. It occurs when people who are working or visiting the jungle get bitten by an infectious mosquito. (ii) The intermediate or savanna cycle is typically found in Africa and involves the spread of viruses from mosquitos to humans who live or work along jungle borders. In this transmission cycle, the virus can be spread from monkeys to humans via mosquitos. (iii) The urban transmission cycle involves a viraemic person who got the virus in the jungle or intermediate cycle and then returned to an urban location. Humans may develop high viremia throughout the process and infect mosquitos, which can then transfer the virus to other humans in metropolitan settings.^10^ The urban cycle may have *A. aegypti* or *Aedes bromeliae* as vectors. However, human‐to‐human transmission is not possible and requires the presence of an infected vector.^11^, ^12^ This extrapolates that rather than the monkey–mosquito–monkey YF cycle, in which humans are unintentional hosts, the virus during outbreak may spread through an urban YF cycle, in which human‐to‐human transmission occurs through mosquito bites.^9^ This clearly demonstrates that YF is a major worldwide challenge that calls for fresh approaches to strategy. The Eliminate Yellow Fever Epidemics (EYE) Strategy was created in response to the growing risk of YF urban outbreaks spreading internationally. EYE is led by WHO, UNICEF, and GAVI, the Vaccine Alliance, and it supports 40 countries and over 50 partners.^13^

The past COVID‐19 pandemic has severely hampered the economic development of numerous nations, including Angola,^14^, ^15^ there is no doubt that the impact of the country's overburdened healthcare system on the surveillance of numerous arbovirus diseases, including YF, has grown to be a serious concern for health officials.

Because there is little information available regarding the last YF outbreak in Angola, we aimed to perform a retrospective study on this outbreak, where there was significant mortality. This demonstrates the features and lessons learned from the historical YF epidemic in Angola and helps the authorities improve their health policy guidelines. Therefore, it is worth reflecting on this outbreak because of the impact of climate change on current and future burdens caused by re‐emerging viruses, including YFV.

## 2 METHODS

### 2.1 Reporting of YF cases

The YF epidemic began in December 2015 in the peri‐urban municipality of Viana (Km 30) of Luanda Province with the occurrence of four cases of Eritrean citizens that resulted in death (Supporting Information S1: Figure 2). Subsequently, new cases emerged with a rapid progression of the epidemic in the municipalities of Luanda and later in Huíla, Huambo, and the other provinces. The Ministry of Health of Angola mandated the Direcção Nacional de Saúde Pública (DNSP) to immediately record all symptomatic reported YF cases. Nevertheless, fever and jaundice were accompanied by at least one of the following symptoms: headache, asthenia conjunctivitis, vomiting, or no apparent other cause were the initial case definitions for YF. Other symptoms, such as hemorrhagic manifestations, that had different types of hemorrhage were considered.

### 2.2 Public health laboratory surveillance in Angola

In Angola, the national public health laboratory surveillance program for notifiable conditions, including YF in all age groups is managed and conducted by INIS. Information (including laboratory surveillance) from public and private healthcare facilities is collected, validated, and sent to the public health national surveillance system. The typical reporting time from municipal healthcare facilities to the first reporting of the result was roughly 1 week. The study period for this report began on December 5, 2015, to December 23, 2016. All cases that were identified before the epidemic (before epidemiological Week 49) are not included in this study because, although routine vaccination was implemented, especially for travelers, before that, YF was not consistently monitored by the public health surveillance system. In this report, we considered YF cases, all patients or patient samples that were submitted to the National Diseases Surveillance Department with a suspicion of YF, and with or without laboratory confirmation. We defined laboratory‐confirmed YF cases as samples of patients sent to the INIS characterized by the presence of clinical symptoms of YF and an IgM serological test result positive for YFV or a result positive for YFV RNA by means of a reverse transcription polymerase chain reaction (RT‐PCR) assay. This study was carried out in compliance with the Helsinki Declaration, as amended by the 64th World Medical Association General Assembly in Fortaleza, Federative Republic of Brazil, in October 2013; and informed consent was acquired from all subjects and/or their legal guardian(s) in case of minor subject. Thereafter, we solicited the ethics approval from the independent Ethics Review Board of the Faculty of Medicine of the Universidade Agostinho Neto, Luanda, Angola, before the commencement of the experiment with reference No. 21/2022.

### 2.3 Geographic distribution of the epidemic

**Figure 1:**
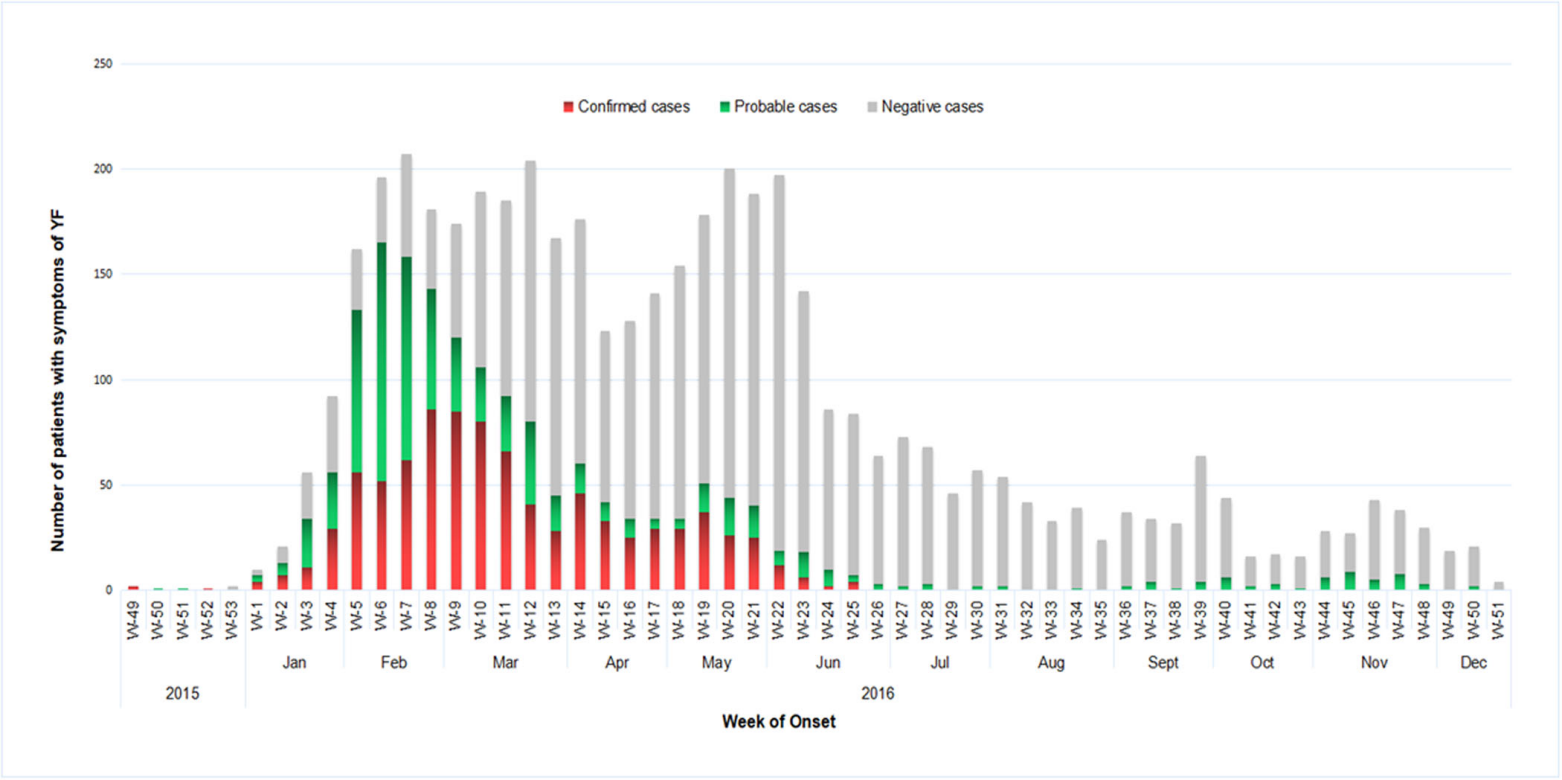
The epidemiological curve of confirmed, suspected, and negative cases of YF in Angola, December 5, 2015, to December 23, 2016. Red bar = confirmed cases; green bar = probable cases; and gray bar = negative cases. YF, yellow fever.

Suspected cases of YF had a notification form that included demographic information, such as age, gender, and date of beginning of symptoms. We investigated the incidence of reported YF for both the total population and positive patients according to the reporting area (16 out of 18 provinces, which makes 85 municipalities) to estimate the geographic dispersion of the outbreak, as shown in Supporting Information S1: Figure 1. Furthermore, we computed the cumulative incidence of YF in the overall population and the male‐to‐female incidence ratio by age and sex per 100,000 individuals using the number of YF cases and population estimates.

### 2.4 IgM enzyme‐linked immunosorbent assay (ELISA) serological test of YF

A total of 4618 blood samples from suspected YF cases were referred to the national laboratory of INIS between December 5, 2015, and December 23, 2016. Blood samples were sent from 16 provinces in Angola. During the epidemic period, serological tests for YF were performed at the referral national laboratory of INIS with the use of ELISA assay. We adapted the IgM ELISA as described by Martin et al.^16^ For the individual detection of anti‐YFV IgM by using sera of suspected patients with YF in optimal dilutions as established by antigen titration and the use of the 6B6C‐1‐HRP peroxidase‐conjugated monoclonal antibody (CDC) or TMB (3,3′,5,5′‐Tetramethylbenzidine) substrate (KPL) in a 1:100 dilution. A spectrophotometer was used to measure the optical density (OD) at a wavelength of 405–650 nm, or 450–650 nm when TBM was used.

To stop the reaction, 100 mL of 1% SDS (sodium dodecyl sulfate) was added. When a cross‐reactive sample showed at least a twofold rise in titer, it was deemed positive; otherwise, the result suggested a potential cross‐reaction between flaviviruses.^17^, ^18^

### 2.5 Molecular detection of YFV

After being analyzed using ELISA assay, the samples were confirmed with laboratory molecular test. For the confirmatory test, we performed the RT‐PCR assay, which consisted of testing YFV by using RT‐PCR as advised by the World Health Organization (WHO) using the method previously demonstrated by Domingo et al.^19^ Briefly, the samples were then tested by pan‐flavivirus RT‐PCR targeting the flavivirus YFV 5′ UTR region using primers. To perform the molecular detection of YFV, we utilized the AgPath‐IDTM One‐Step RT‐PCR System AB Applied Biosystems (7500 Fast Real‐Time PCR System), 100rxn reference: AM1005. We used oligonucleotides (5′‐FAM probe) in a 25 μL final reaction volume according to the Domingo and Colleagues protocol.^19^ The test has a sensitivity of 25 genomic copies for each reaction. Using ABI7500 equipment (ThermoFisher Scientific), the thermal profile included reverse transcription at 50°C for 20 min, enzyme activation at 95°C for 5 min, and 45 cycles for hybridization and extension at 95°C for 15 s and 60°C for 45 min. The following primers and probes were used in this study:

Primer YFall Forward: 5′‐GCTAATTGAGGTGYATTGGTCTGC‐3′;

Primer YFall Reverse: 5′‐CTGCTAATCGCTCAAMGAACG‐3′;

Probe YFall: 5′‐FAM‐ATCGAGTTGCTAGGCAATAAACAC‐TMR‐3′.

### 2.6 Vaccination coverage

One of the main measures to prevent and control YF is massive vaccination, and this should be prioritized in areas with evidence of virus circulation. Here, we aimed to assess the vaccination coverage during the YF epidemic in all provinces. YF vaccination coverage rate was assessed as the ratio between the number of people vaccinated for a defined target over a given period and the number of people in the reference population for this same target over the study period. The *χ* ^2^ test was used to examine differences in the proportions of people vaccinated.

### 2.7 Statistical analysis

For all measures of disease burden and mortality, data were presented as absolute counts, cases per 100,000 population, and incidence (cases of new disease per 100,000 population per age group). Using age and sex as the primary variables, we estimated the incidence ratios comparing male and female patients using Poisson regression along with 95% confidence intervals (CIs). Only laboratory‐confirmed cases were used in the analyses, which were carried out as part of national public health practice and summarized data from continuing epidemic surveillance. Data were analyzed using Proc Genmod software (SAS), and the *χ* ^2^ test was used to examine differences in the proportions of people vaccinated.

## 3 RESULTS

### 3.1 Patients reported with YF

From December 5, 2015, through December 23, 2016, a total of 4618 suspected YF cases were documented in Angola, with at least 884 (19.1%) being laboratory‐positive by ELISA and RT‐PCR assays. The remaining 80.9% of laboratory‐negative cases were still classified as suspected cases. During this period, 384 deaths related to YF were reported in Angola. The average case fatality rate was 13.6 among laboratory‐confirmed YF cases. At the time the case was recorded, every patient who had been diagnosed with YF was admitted to the hospital. The number of reported YF cases steadily increased from Week 3 of 2016 through Week 22 of 2016, with February through March 2016 having the highest number of incidents recorded (Figure 1). Overall fewer occurrences were reported, while some regions reported a slight increase in cases during April and May 2016 (Weeks 14 through 21).

### 3.2 Extent of YF outbreak in Angola

**Figure 2:**
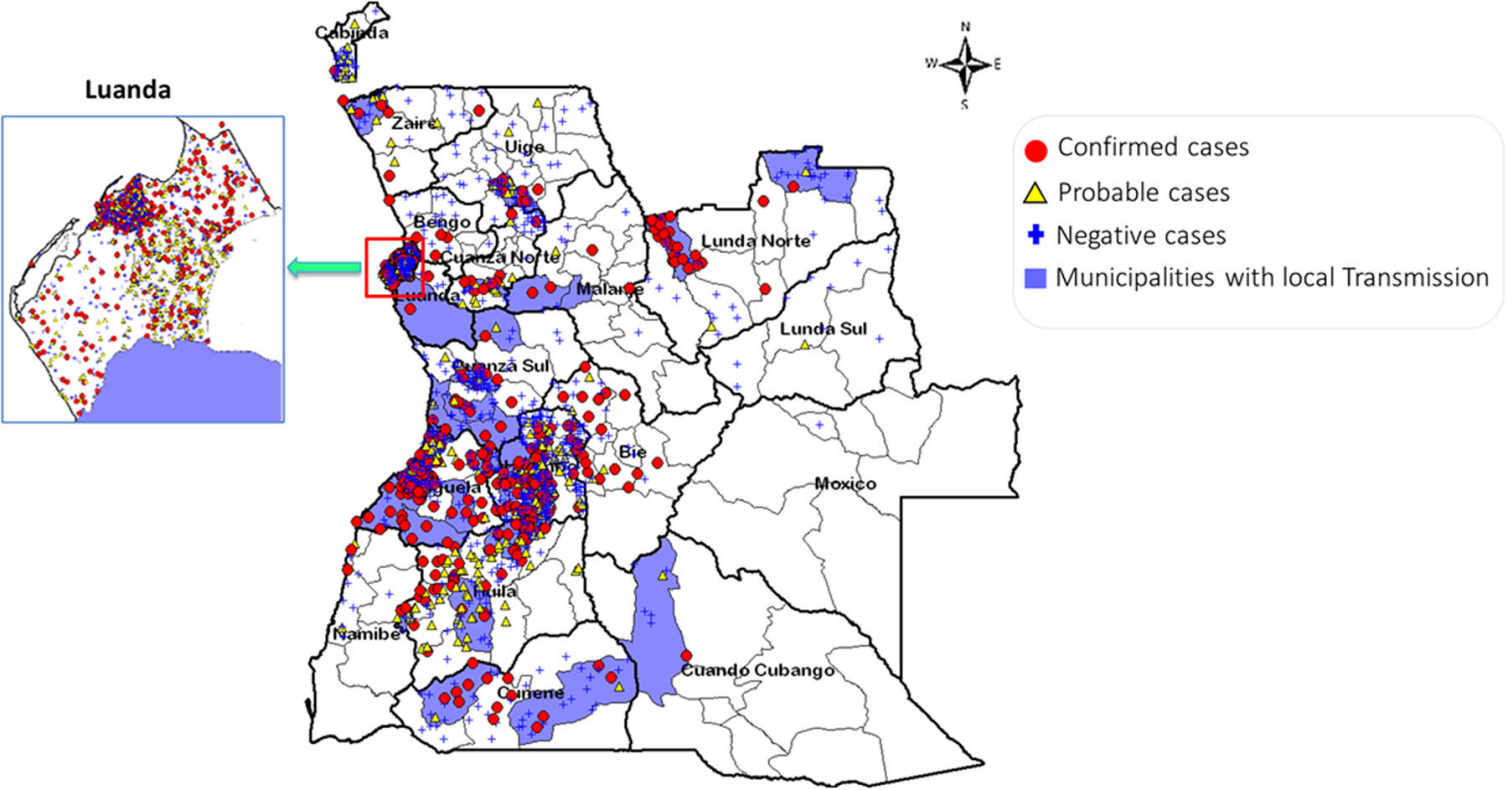
Geographical distribution of yellow fever cases in Angola.

**Figure 3:**
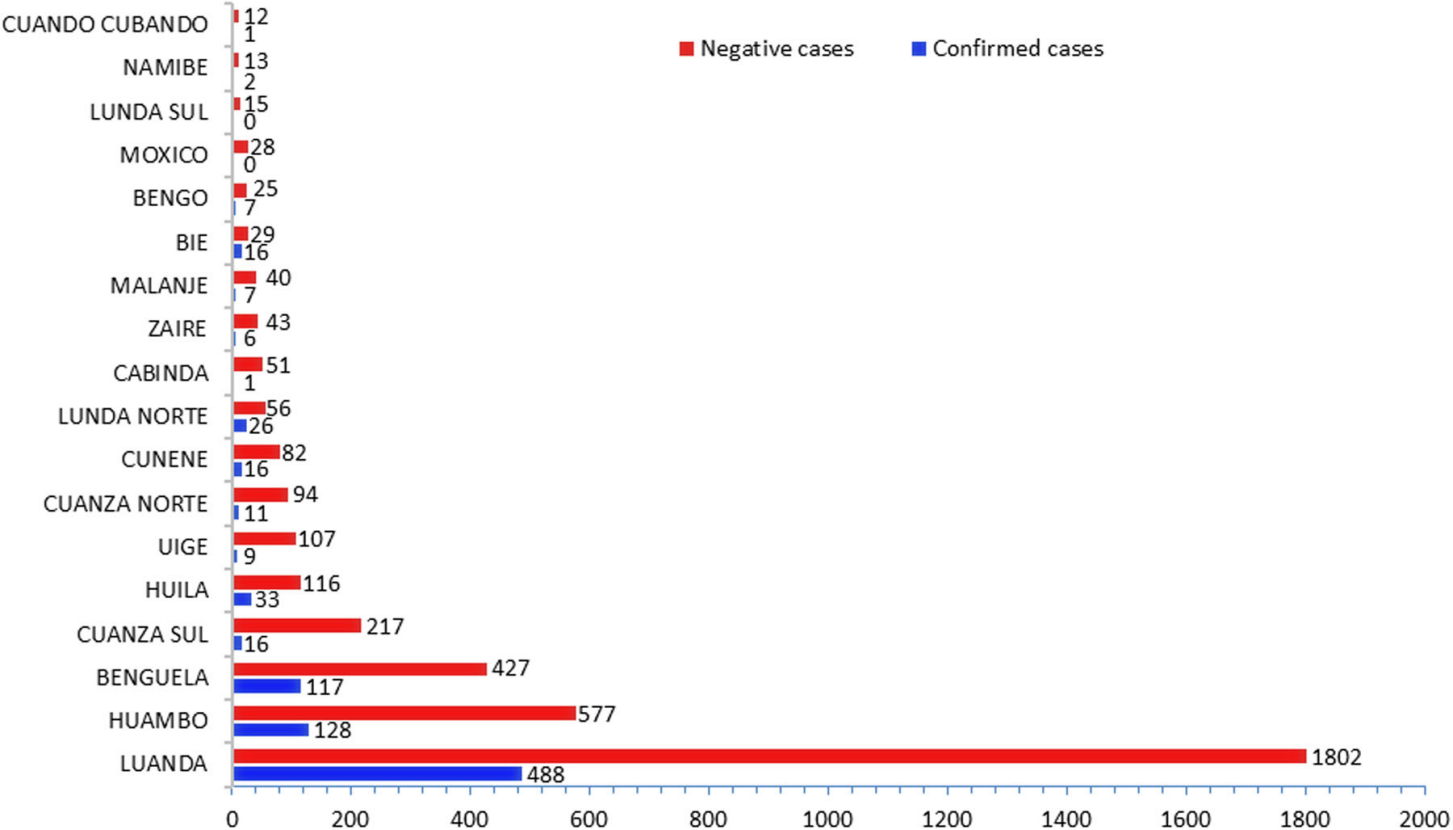
Bar plot showing the total number of confirmed cases of yellow fever by province.

The YFV has spread quickly throughout Angola since the initial cases that were proven to have been acquired locally. With at least one laboratory‐confirmed case in each of the 16 provinces that have reported, the YF cases are widely dispersed throughout Angola. Local transmission was reported in 12 provinces: Benguela (17 cases), Cuando Cubango (one case), Cuanza Norte (two cases), Zaire (one case), Cuanza Sul (eight cases), Cunene (four cases), Huambo (17 cases), Huíla (five cases), Luanda (one case), Luanda Norte (five cases), Malanje (two cases), and Uíge with four cases (Figure 2). The capital city of Angola, Luanda, is the most populated city and was the epicenter of the epidemic with 488 confirmed cases, followed by Huambo province (128 cases) and Benguela province (with 117 cases). Except for the Lunda Sul and Moxico provinces that were not affected, the rest of the provinces have less than 35 confirmed cases (Figure 3).

### 3.3 Incidence ratio by sex

Male patients were diagnosed with YF in about two‐thirds of the reported cases. However, the predicted incidence varied significantly depending on age and gender. We observed that the incidence was approximately three times higher in male patients (*n* = 223; 10.9%) than in female patients (*n* = 59; 2.6%) in the 20–29 age group, followed by the age group 10–19 with *n* = 211 (6.8%) in males versus *n* = 108 (3.3%) in females, and the age group 30–39 with *n* = 68 (4.8%) in males compared to females (*n* = 28; 1.8%) (Table 1). The other groups reported an incidence below 3.0% (Table 1). A similar incidence or no case of YF was reported in senior citizens aged 70 or older, in male and female patients.

**Table 1:** Incidence of YF according to sex and age (December 5, 2015–December 23, 2016).

Age	Male sex			Female sex			Female‐to‐male incidence ratio (95% CI)
	Population	YF cases	Incidence per 100,000	Population	YF cases	Incidence per 100,000	
		Number			Number		
All ages	13,355,101	617	4.62	14,148,475	267	1.89	0.41 (0.37–0.45)
Ages group (year)							
0–9	4,711,070	74	1.57	4,767,481	55	1.15	0.73 (0.68–0.79)
10–19	3,111,622	211	6.78	3,231,359	108	3.34	0.49 (0.45–0.54)
20–29	2,050,166	223	10.88	2,226,578	59	2.65	0.24 (0.21–0.28)
30–39	1,427,568	68	4.76	1,589,854	28	1.76	0.37 (0.33–0.41)
40–49	971,944	24	2.47	1,069,114	12	1.12	0.45 (0.41–0.50)
50–59	599,467	14	2.34	667,241	4	0.60	0.26 (0.22–0.29)
60–69	311,345	3	0.96	366,569	1	0.27	0.28 (0.25–0.32)
70+	171,919	0	0.00	230,279	0	0.00	0.00 (0.00–0.00)

Abbreviations: CI, confidence interval; YF, yellow fever.

### 3.4 Clinical characteristics of confirmed patients with YF in Angola during the period from December 2015 through December 2016

All 884 confirmed patients with YF provided information regarding signs, symptoms, and exposures. The most reported signs or symptoms were fever (measured or reported) and jaundice, accounting for 100% (Table 2). Other symptoms included headache (77.5%), asthenia (77.1%), vomiting (61.8%), hemorrhagic manifestations (19.1%), and other types of hemorrhage (12.4%). The median duration of fever, headache, and vomiting was 3–4 days (range, 2–14). With the reappearance of fever and other symptoms such as asthenia, vomiting, hemorrhagic manifestations, and other types of hemorrhages that impair the function of various organisms and lead patients to enter the toxic phase of the disease, most of these patients died within 7–10 days (Table 2).

**Table 2:** Clinical characteristics of patients with confirmed yellow fever in Angola during the period from December 2015 through December 2016.

Signs or symptoms	No. of patients (%)
Fever^a^	884 (100)
Jaundice	884 (100)
Headache	685 (77.5)
Asthenia	682 (77.1)
Vomiting	546 (61.8)
Hemorrhagic manifestations	169 (19.1)
Types of hemorrhage (total 94)	(12.4)
Gingivorrhagia	27
Hematemesis	23
Epistaxis	23
Melena	10
Conjunctival	3
Gingivorrhagia/epistaxis	3
Vaginal	2
Melena/hematemesis	1
Gingivorrhagia/hematuria	1
Conjunctival/epistaxis	1

^a^ Cases of measured and subjective fever are included.

### 3.5 Vaccine coverage and protective immunity in the overall population

**Figure 4:**
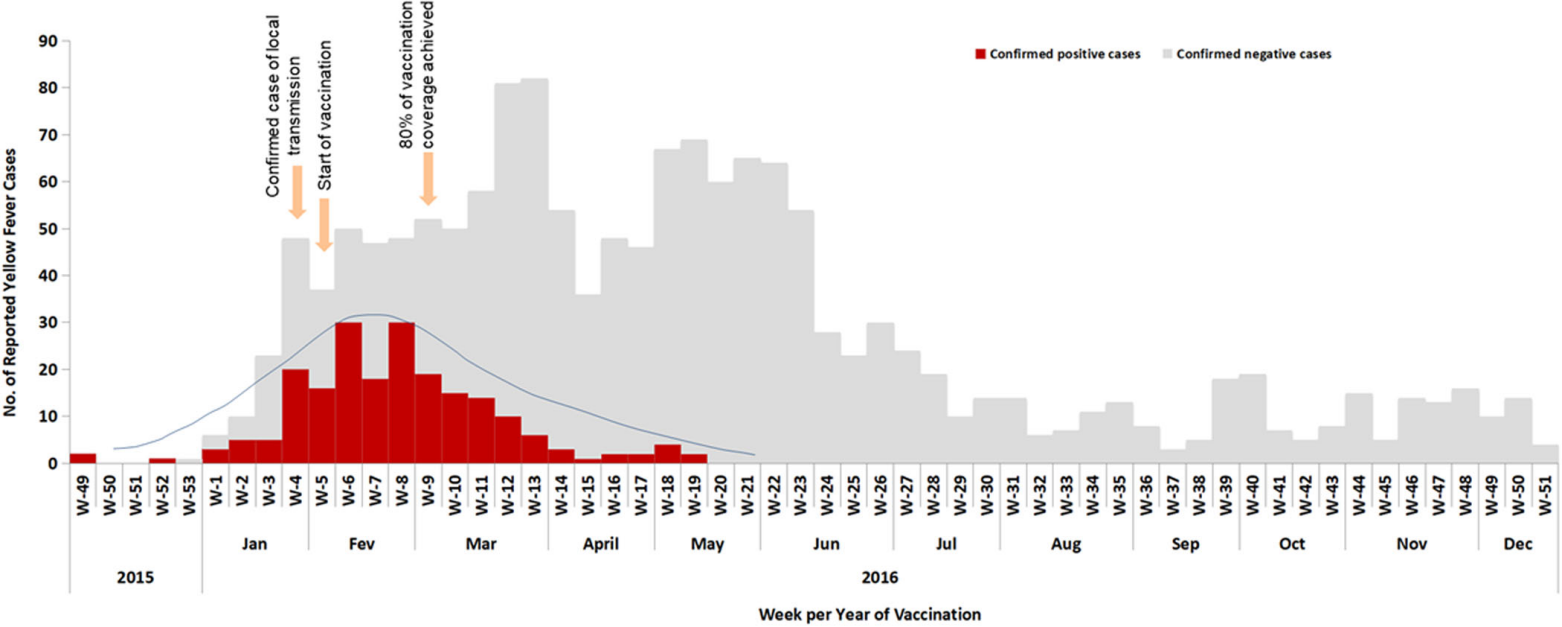
Yellow fever vaccination coverage in the overall population.

The Angolan Ministry of Health, at the time, strategized to achieve 95% or more coverage for YF vaccination in the target population over 6 months' time. This strategy served to interrupt the transmission of the YFV through massive vaccination of the population in the shortest possible time. Of the 18 provinces of Angola that were vaccinated for the YF vaccine, 11 met the 95% coverage target set by the government, with Uíge (113%) being the most covered province, followed by Moxico (110%), Cuando Cubango and Huíla (both with 103%), Lunda Sul (102%), Cabinda and Luanda Norte (101%), Namibe and Benguela (100%), and 96% target coverage accounted for the province of Bié. However, Bengo and Luanda did not achieve the 95% target coverage set by the Angolan government, they reported 80% and 93% of vaccination coverage achievement, respectively. Overall, the 95% coverage target was achieved for the entire country (Supporting Information S1: Table 1). The over 100% coverage observed in some provinces could be due to difficulties with population estimates. To assess the vaccine response in the Angolan population, Viana, a municipality in Luanda that is the most populated municipality in Luanda, was used as a priority and experimental site. In this municipality, participants started the YF vaccination at Week 5 (early February 2016). After reaching the 80% target coverage, a decreased trend of confirmed cases was evident until surpassing the baseline (Figure 4), suggesting that the vaccine is an important tool for controlling infectious disease epidemiology.

### 3.6 Case fatality ratio for YF during the period from December 2015 through December 2016

Within 12 months, the cumulative incidence of death in male patients with YF per 100,000 inhabitants was 0.64%, and in female patients with the same cause, it was 0.25%. When comparing the female‐to‐male death incidence ratio, we found 0.38% (95% CI, 0.34–0.43). In relation to age, the female‐to‐male incidence ratio was higher in the age group 10–19, with the highest 0.84% (95% CI, 0.78–0.90), followed by the age group 20–29 with the female‐to‐male incidence ratio of 0.77% (95% CI, 0.71–0.83), then the age group 60–69 with 0.45% (95% CI, 0.41–0.50), thereafter the age group 0–9 with 0.23% (95% CI, 0.20–0.26), and finally the age groups 20–29 and 50–59 with 0.19% (the 95% CI was 0.16–0.22) and 0.18% (the 95% CI was 0.15–0.21), respectively (Table 3). No case was reported in the age groups 60–69 and 70 or older.

**Table 3:** Number of confirmed deaths by YF according to sex and age group during the period from December 2015 through December 2016.

Age	Male sex			Female sex			Female‐to‐male incidence ratio (95% CI)
	Population	YF cases	Incidence per 100,000	Population	YF cases	Incidence per 100,000	
		Number			Number		
All ages	13,355,101	86	0.64	14,148,475	35	0.25	0.38 (0.34–0.43)
Ages group (year)							
0–9	4,711,070	13	0.28	4,767,481	3	0.06	0.23 (0.20–0.26)
10–19	3,111,622	16	0.51	3,231,359	14	0.43	0.84 (0.78–0.90)
20–29	2,050,166	39	1.90	2,226,578	8	0.36	0.19 (0.16–0.22)
30–39	1,427,568	7	0.49	1,589,854	6	0.38	0.77 (0.71–0.83)
40–49	971,944	6	0.62	1,069,114	3	0.28	0.45 (0.41–0.50)
50–59	599,467	5	0.83	667,241	1	0.15	0.18 (0.15–0.21)
60–69	311,345	0	0.00	366,569	0	0.00	0.00 (0.00–0.00)
70+	171,919	0	0.00	230,279	0	0.00	0.00 (0.00–0.00)

Abbreviations: CI, confidence interval; YF, yellow fever.

## 4 DISCUSSION

The recent outbreak of YF has spread rapidly throughout Angola and other countries, after the first group of laboratory‐confirmed cases was reported in December 2015. Of the total of 4618 cases reported, 884 were confirmed positive, including male (*n* = 617) and female (*n* = 267) cases from December 5, 2015, through December 23, 2016. However, because they do not take into consideration infections that do not cause symptoms or clinical illnesses that go unreported, these figures understate the overall impact of the YF outbreak. In comparison to their female counterparts, males between the ages of 20 and 29 had a higher incidence of YF (10.88%). The extent to which testing and reporting biases may be exaggerating the number of male patients with YF is unclear. We observed higher incidence of YF among males between the age groups than among females in the same age group, this could be the outcome of a real rise in risk as exposure to mosquitoes is higher in males because they work outdoors, or reporting or testing bias, or the fact that females are more likely to be vaccinated. However, individuals between the ages of 10–19 years, 30–39 years, and 40–49 years showed higher YF incidence in men than among women, suggesting that men may be more susceptible to symptomatic YFV infection, because in this age group, a reporting bias based on worry about social behaviors (smoking and alcoholism) would have a higher role.

The highest mortality rates were reported in young adult male subjects and female teenage subjects. Our result agrees with the previously reported data from other regions.^20^, ^21^ Surprisingly, no mortality cases were reported in the elderly among both male and female subjects. The immunity observed in the elderly could be due to the one‐dose vaccination that may give a lifelong immunity,^22^ or these individuals could have had prior exposure to mosquitoes and viruses and thus have some infection‐acquired immunity.

People living in distant and remote area, porous borders with neighboring countries, and underreporting cases by healthcare practitioners are further obstacles to the continued population‐based surveillance of YF. These factors make it more difficult to identify all cases of YF. It is obvious that we may have missed or underreported some cases if they happen in regions that are not thought to be YF‐prone, such as those that are higher than 2000 meters above sea level. Geographic variation in cases of YF is correlated with Angolan areas with high concentrations of *A. aegypti* vectors. According to the WHO, an individual is considered to be a YF‐suspect if he exhibits acute feverish illness and develops jaundice within 14 days of the onset of symptoms.^23^ The clinical signs and symptoms of YFV infection were consistent with those described in previously published reports.^3^, ^24^, ^25^

Moreover, changing the definitions of YF cases may have impacted the reporting of YV cases throughout the study period, and several patients who presented with afebrile YF may not have been reported. Angola still has a weak public healthcare system, consequently, it is uncertain if reporting regions strictly followed prescribed symptom criteria while sending samples of YF cases to the INIS. Also, since the symptoms specified in the case definition overlap with other disorders such as dengue viral sickness, chikungunya, malaria, and hepatitis, there may be differential reporting of clinically compatible cases.^26^, ^27^ Health providers and the National Public Health Surveillance Department are in a unique position to educate patients about the importance of mosquito bite prevention to reduce the risk of all mosquito‐borne illnesses.^28^ Since YF is traditionally transmitted through the cycle of monkey–mosquito–monkey, there was an observation of the rapid spread of YF cases in Angola through neighboring country Congo‐Kinshasa, It implies that, rather than the conventional YF cycle, the transmission may have happened through an urban YF cycle, in which the virus spreads between people by the bite of an *A. aegypti* mosquito. If this has happened, therefore, a serious global threat should be considered, and existing EYE strategies need to be better implemented. It is worth mentioning that previous studies showed that when viral RNA was no longer found in sera, YFV RNA could still be detected in urine samples.^29^, ^30^, ^31^ Further studies should also be conducted to evaluate whether YF is sexually transmitted. In Angola, a nationwide population‐based surveillance is crucial for monitoring the outbreak's status by focusing on preventative activities and swiftly analyzing adverse outcomes. Despite its under‐resourced health system, the outbreak was rapidly identified, prompting cordon vaccination in Luanda in early February 2016, followed by mass immunization throughout the country.^32^ The overall immunization target for the whole country, as decided by the national authority, was achieved by reaching the target of 95%. However, Benguela, Bié, Cabinda, Cuando Cubango, Huíla, Luanda Norte, Lunda Sul, Namibe, Moxico, and Uíge were the provinces to achieve the target set by the authority. Other provinces had a 95% target below. Luanda city was the epicenter of the epidemic, and Viana municipality is where the outbreak started. The National Public Health Surveillance Department and INIS must intensify disease surveillance by implementing laboratory surveillance and promoting prevention measures such as vaccination for YF by ensuring good coverage, as well as vector control activities. The resurgence of YF cases in Africa, and particularly in Angola, can be ascribed to a number of factors, including the virus's sylvatic life cycle and intermittent mass vaccination, particularly in politically and economically fragile regions.^1^ It is worth mentioning that, before the 2015–2016 YF outbreak in Angola, an estimated 52.0% of children in Angola, both male and female, did not have any vaccination records.^3^ Some factors that may interfere with vaccination coverage include (i) a lack of sustainability of the immunization system, (ii) population knowledge and attitudes about vaccination, and (iii) a lack of communication and information.

Our study has several limitations. First, we did not assess the national seroprevalence of YF to determine the overall immunity in the vaccinated population, also, we do not have data about vaccine effectiveness in the studied population that could enable us to investigate whether those who died had received a YF vaccine. Second, we did not perform the genomic analysis to determine the genotype and phylodynamic of the YFV. Third, we did not investigate to confirm the genus of vector responsible for transmitting the YFV. Although data is from more than 5 years ago, we believe that it represents a global conceptual advance.

## 5 CONCLUSION

This study demonstrates features of the YF epidemic that occurred in Angola. Also, it demonstrates that YF causes deaths in young people, but vaccination appeared to bring the outbreak to an end. Therefore, to reduce the chance of reemerging human infections, laboratory and public health surveillance systems need to be reinforced.

## AUTHOR CONTRIBUTIONS

The authors consent to participate in peer review and consent to publish. All authors have read and approved the final version of the manuscript. Ngiambudulu M. Francisco had full access to all the data in this study and takes complete responsibility for the integrity of the data and the accuracy of the data analysis.

## CONFLICT OF INTEREST STATEMENT

The authors declare no conflict of interest.

## TRANSPARENCY STATEMENT

The lead author Ngiambudulu M. Francisco affirms that this manuscript is an honest, accurate, and transparent account of the study being reported; that no important aspects of the study have been omitted; and that any discrepancies from the study as planned (and, if relevant, registered) have been explained.

## Supporting information

Supporting information.

## Data Availability

All data generated or analyzed during this study are included in this published article (and its supplementary information files).
