# Supplementary material for: Assessment of the yellow fever outbreak in Angola from December 2015 through December 2016: A retrospective study
Source: Health Sci Rep. 2024 Feb 15;7(2):e1924. doi: 10.1002/hsr2.1924 (PMC10913757; doi:10.1002/hsr2.1924)
Supplement: Supplementary file 1 — Supporting information. [file HSR2-7-e1924-s001.docx]

**Supplement Figures**

**
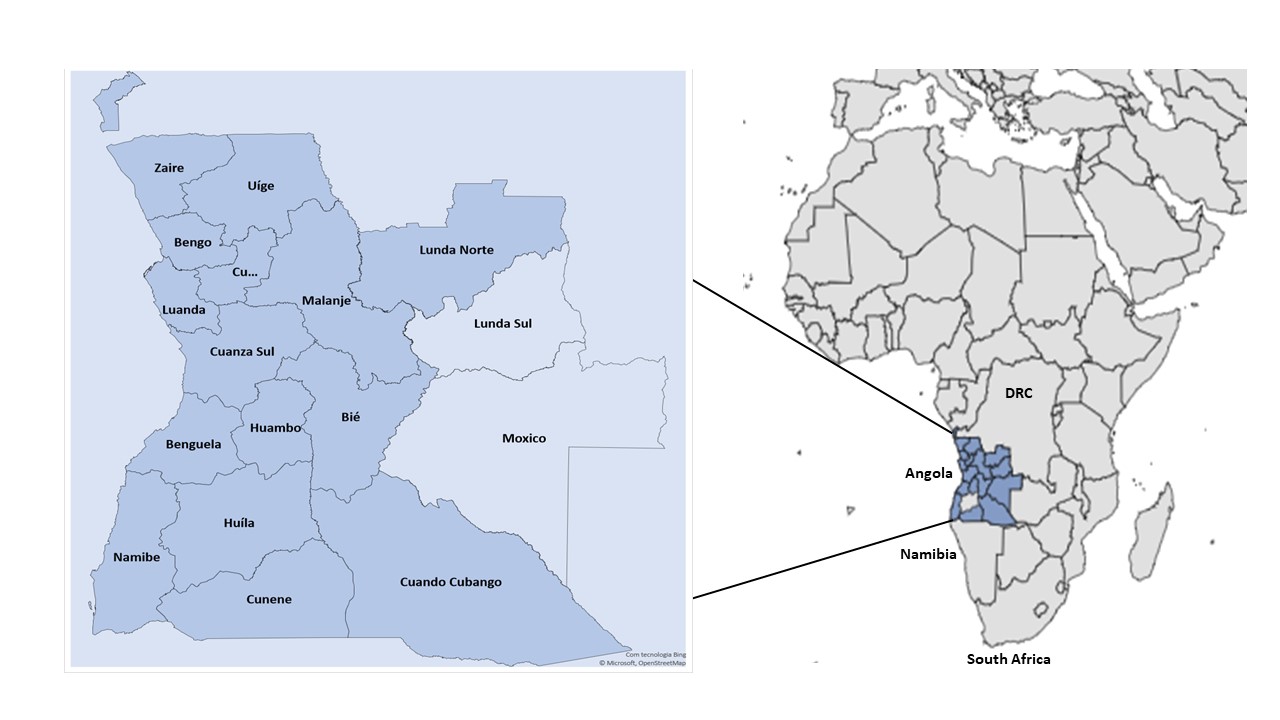
**

**Supp. Figure 1: Distribution of provinces affected by the Yellow Fever outbreak in Angola from December 2015 until December 2016**


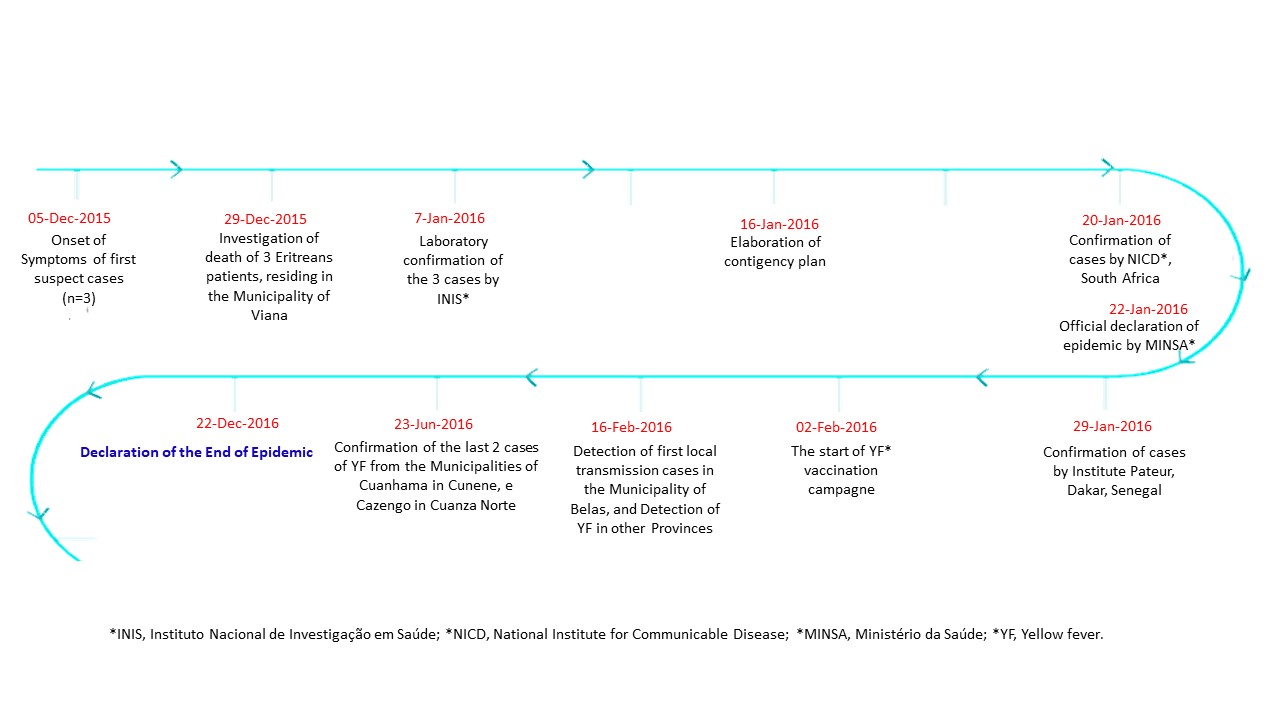


**Supp. Figure 2: Milestones of the Yellow Fever Public Health Response in Angola**

**Supplement Tables**

**Supplement Table 1. Vaccine coverage in the overall population**

| **Province** | **Targeted population** | **Launching date** | **Population vaccinated** | **Administrative coverage (%)** |
| --- | --- | --- | --- | --- |
| Belas | 1,071,662 | 19-Feb-2016 | 1,292,404 | **121%** |
| Cacuaco | 887,829 | 29-Feb-2016 | 776,075 | **87%** |
| Cazenga | 867,659 | 29-Feb-2016 | 825,793 | **95%** |
| Icolo e Bengo | 75,103 | 28-Mar-2016 | 46,309 | **62%** |
| Ingombota | 89,556 | 24-Mar-2016 | 65,083 | **73%** |
| Kilamba Kiaxi | 640,006 | 10-Mar-2016 | 241,812 | **38%** |
| Maianga | 660,884 | 14-Mar-2016 | 489,998 | **74%** |
| Quissama | 25,240 | 28-Mar-2016 | 15,572 | **62%** |
| Rangel | 136,031 | 28-Mar-2016 | 48,021 | **35%** |
| Samba | 160,174 | 24-Mar-2016 | 58,535 | **37%** |
| Sambizanga | 433,970 | 20-Mar-2016 | 146,149 | **34%** |
| Viana | 1,535,102 | 02-Feb-2016 | 2,130,549 | **139%** |
| **Luanda** | **6,583,216** |  | **6,136,300** | **93%** |
| Baia Farta | 103,623 | 16-May-2016 | 105,116 | **101%** |
| Balombo | 99,932 | 16-May-2016 | 101,700 | **102%** |
| Benguela | 598,902 | 12-Apr-2016 | 563,250 | **94%** |
| Bocoio | 155,397 | 30-Jun-2016 | 138,747 | **89%** |
| Caimbambo | 81,212 | 15-Oct-2016 | 81,809 | **101%** |
| Catumbela | 173,601 | 13-Apr-2016 | 188,421 | **109%** |
| Chongoroi | 81,977 | 30-Jun-2016 | 85,175 | **104%** |
| Cubal | 289,703 | 16-May-2016 | 297,264 | **103%** |
| Ganda | 226,051 | 15-Oct-2016 | 227,923 | **101%** |
| Lobito | 335,601 | 13-Apr-2016 | 356,144 | **106%** |
| **Benguela** | **2,145,999** |  | **2,145,549** | **100%** |
| Dande | 219,270 | 15-Oct-2016 | 175,351 | **80%** |
| **Bengo** | **219,270** |  | **175,351** | **80%** |
| Cuito | 426,780 | 15-Oct-2016 | 411,357 | **96%** |
| **Bie** | **426,780** |  | **411,357** | **96%** |
| Belize | 19,574 | 17-Aug-2016 | 21,780 | **111%** |
| Buco Zau | 34,051 | 17-Aug-2016 | 33,000 | **97%** |
| Cabinda | 601,892 | 17-Aug-2016 | 603,328 | **100%** |
| Cacongo | 37,004 | 17-Aug-2016 | 38,877 | **105%** |
| **Cabinda** | **692,521** |  | **696,985** | **101%** |
| Menongue | 308,509 | 17-Aug-2016 | 331,991 | **108%** |
| Calai | 20,266 | 08-Aug-2016 | 16,973 | **84%** |
| Dirico | 14,691 | 08-Aug-2016 | 8,939 | **61%** |
| Cuangar | 27,503 | 08-Aug-2016 | 23,339 | **85%** |
| **Cuango Cubango** | **370,969** |  | **381,242** | **103%** |
| Ambaca | 61,209 | 15-Oct-2016 | 51,697 | **84%** |
| Cazengo | 166,860 | 30-Jun-2016 | 142,561 | **85%** |
| Cambambe | 89,498 | 17-Aug-2016 | 74,639 | **83%** |
| **Cuanza Norte** | **256,358** |  | **217,200** | **85%** |
| Amboim | 236,339 | 16-May-2016 | 228,060 | **96%** |
| Cassongue | 145,579 | 16-May-2016 | 135,907 | **93%** |
| Conda | 90,234 | 15-Oct-2016 | 93,776 | **104%** |
| Ebo | 159,024 | 16-May-2016 | 159,495 | **100%** |
| Libolo | 85,630 | 16-May-2016 | 88,897 | **104%** |
| Porto Amboim | 120479 | 15-Oct-2016 | 99,640 | **83%** |
| Seles | 176,058 | 16-May-2016 | 170,883 | **97%** |
| Sumbe | 269,341 | 17-Aug-2016 | 191,089 | **71%** |
| **Cuanza Sul** | **1,282,684** |  | **1,167,747** | **91%** |
| Cahama | 69,519 | 22-Jun-2016 | 65,132 | **94%** |
| Cuanhama | 362,710 | 22-Jun-2016 | 329,772 | **91%** |
| Namacunde | 144,624 | 17-Agu-2016 | 91,306 | **63%** |
| Ombadja | 291,862 | 15-Oct-2016 | 251,464 | **86%** |
| **Cunene** | **868,715** |  | **737,674** | **85%** |
| Bailundo | 283,887 | 16-May-2016 | 270,735 | **95%** |
| Caala | 268,734 | 13-Apr-2016 | 222,811 | **83%** |
| Ecunha | 79,334 | 16-May-2016 | 75,022 | **95%** |
| Huambo | 689,301 | 13-Apr-2016 | 558,150 | **81%** |
| Londuimbale | 125,214 | 02-Jul-2016 | 114,709 | **92%** |
| Longonjo | 87,329 | 02-Jul-2016 | 88,892 | **102%** |
| Ukuma | 42,950 | 16-May-2016 | 47,348 | **110%** |
| Tchinjenje | 28,371 | 17-Aug-2016 | 28,909 | **102%** |
| **Huambo** | **1,605,120** |  | **1,406,576** | **88%** |
| Caconda | 160,892 | 16-May-2016 | 167,102 | **104%** |
| Gambos | 76,456 | 29-Jun-2016 | 81,982 | **107%** |
| Humpata | 83,267 | 28-Jun-2016 | 83,731 | **101%** |
| Lubango | 736,077 | 17-Aug-2016 | 737,475 | **100%** |
| Quipungo | 147,818 | 16-May-2016 | 168,781 | **114%** |
| **Huila** | **1,204,510** |  | **1,239,071** | **103%** |
| Capenda Camulemba | 54,098 | 02-Jul-2016 | 46,528 | **86%** |
| Caungula | 27,266 | 02-Jul-2016 | 20,967 | **77%** |
| Cambulo | 113,570 | 17-Aug-2016 | 110,714 | **97%** |
| Chitato | 196,869 | 11-Jun-2016 | 203,794 | **104%** |
| Cuango | 174,429 | 4-Jun-2016 | 187,504 | **107%** |
| Cuilo | 19,841 | 01-Jul-2016 | 17,479 | **88%** |
| Xa Muteba | 52,765 | 15-Oct-2016 | 56,052 | **106%** |
| **Lunda Norte** | **638,838** |  | **643,038** | **101%** |
| Muconda | 35,469 | 17-Aug-2016 | 33,061 | **93%** |
| Saurimo | 426,155 | 15-Oct-2016 | 438,003 | **103%** |
| **Lunda Sul** | **461,624** |  | **471,064** | **102%** |
| Tômbua | 55,211 | 15-Oct-2016 | 55,457 | **100%** |
| **Namibe** | **55,211** |  | **55,457** | **100%** |
| Cacuso | 71,981 | 08-Jul-2016 | 60,852 | **85%** |
| Malanje | 489,867 | 14-Jul-2016 | 396,547 | **81%** |
| Marimba | 27,241 | 17-Aug-2016 | 24,703 | **91%** |
| Massango | 32,811 | 17-Aug-2016 | 33,429 | **102%** |
| **Malange** | **621,900** |  | **515,531** | **83%** |
| Luau | 84,997 | 15-Oct-2016 | 93,476 | **110%** |
| **Moxico** | **84,997** |  | **93,476** | **110%** |
| Maquela do Zombo | 123,073 | 01-Jul-2016 | 117,896 | **96%** |
| Milunga | 48,454 | 17-Aug-2016 | 52,815 | **109%** |
| Negage | 136,323 | 16-May-2016 | 129,732 | **95%** |
| Puri | 35,710 | 23-Jun-2016 | 36,137 | **101%** |
| Quimbele | 130,192 | 17-Aug-2016 | 138,419 | **106%** |
| Uige | 496,567 | 16-May-2016 | 624,138 | **126%** |
| **Uige** | **970,319** |  | **1,099,137** | **113%** |
| Cuimba | 65,011 | 17-Aug-2016 | 58,344 | **90%** |
| Mbanza Congo | 174,920 | 17-ago-2016 | 147,578 | **84%** |
| Noqui | 22,966 | 17-ago-2016 | 22,505 | **98%** |
| Soyo | 219,536 | 16-Jun-2016 | 168,537 | **77%** |
| **Zaire** | **482,433** |  | **396,964** | **82%** |
| **Total of all provinces** | **18,971,464** |  | **17,989,719** | **95%** |
